# Design of osteosynthesis plate for detecting bone union using wire natural frequency

**DOI:** 10.1038/s41598-024-63530-w

**Published:** 2024-05-31

**Authors:** Pisitpong Chancharoen, Pairat Tangpornprasert, Chavarin Amarase, Saran Tantavisut, Chanyaphan Virulsri

**Affiliations:** 1 Center of Excellence for Prosthetic and Orthopedic Implant, Chulalongkorn University, Bangkok, 10330 Thailand; 2 Biomedical Engineering Research Center, Faculty of Engineering, Chulalongkorn University, Bangkok, 10330 Thailand; 3 Department of Mechanical Engineering, Faculty of Engineering, Chulalongkorn University, Bangkok, 10330 Thailand; 4 Hip Fracture Research Unit, Department of Orthopedic, Chulalongkorn University, Bangkok, 10330 Thailand

**Keywords:** Engineering, Biomedical engineering, Mechanical engineering

## Abstract

We have developed a novel osteosynthesis plate with bone union detection using a wire's natural frequency (BUDWF) to provide the quantitative result of bone union detection. The concept for detecting bone union is measuring the rate of frequency change. The frequency is measured from sound generated from the wire attached to a modified plate. The plate is modified from a Syncera ADLER B0409.10 and attached with 0.3 mm diameter 316L stainless steel wire. The sound generation mechanism was created by PEEK and installed on the plate to generate the sound. The preliminary experiments were conducted on a Sawbones tibia composite mimic. We used the cut Sawbones to create fracture samples with a 0, 0.5, 1-, 2-, and 5-mm gap representing the fractured bone with different gap sizes and prepared uncut Sawbones as a union sample. These samples were tested five times, and the sound was recorded from a condenser microphone and analyzed. We found that the BUDWF can differentiate samples with a fracture gap above 2 mm from the union sample, as the differences in the rates of frequency change between samples with a fracture gap above 2 mm and union samples were statistically significant. However, there was a limitation that the BUDWF plate was still unable to differentiate the 0 mm fracture gap and the union sample in this study.

## Introduction

Fracture is the break of continuity of bone caused by applying excessive load. It commonly occurs around the world^1–3^. The cause of fracture is mostly from accidents^4^. Our body has a biological response called fracture healing to regenerate the fractured bone and restore its biomechanical functions^5^. Fixation devices facilitate fracture healing by connecting the fractured bone, sharing applied weight, and maintaining stability^1^. One of the most crucial decisions in fracture healing is determining when a bone is healed^2^. The union bone is ready to resume weight-bearing and start rehabilitation. However, several significant injuries, such as refracture and nonunion, can occur by premature weight bearing^6,7^. The radiograph is the most widely used method for detecting bone union due to its simplicity^2^. The radiograph illustrates the status of the healing process, allowing doctors to assess the fracture and provide appropriate therapies. However, the radiograph result is subjective and based on individual interpretation^6^. Even though there are several score systems for accurately interpreting the fracture radiograph^8,9^, the physicians must have enough experience to score the radiograph correctly^10^, as any misinterpretation might result in delayed or ineffective treatments^7^.

Quantitative results for fracture monitoring can provide helpful information for improving the radiograph's subjective evaluation. Researchers have developed fixation devices with sensors for determining fracture healing to inspect fracture healing with Quantitative results^2,11^. Fracture stiffness is the biomechanical properties of bone related to its strength. It is frequently used to indicate fracture healing as the stiffness increases along with the fracture healing^2,12,13^. The general method for determining fracture stiffness is applying force over the fracture and measuring the displacement or strain by attaching a sensor. The strain gauge was a typical sensor for this approach implemented in internal and external fixation devices^12–16^. For example, Richardson et al.^12^ found that the fracture assessment using their stiffness-measuring external fixator had a lower refracture rate and time to safely remove the fixator than the standard clinical decision, which benefits the patient's well-being. Kienast et al.^15^ effectively demonstrated the strain gauge's ability to detect changes in stiffness in their clinical study. Their study showed a strong correlation between the stiffness changes from the sensor and the CT result, which benefited the fracture healing assessment by providing more information. McGilvray et al.^16^ successfully developed a biocompatible MEMS sensor and used electromagnetic waves to carry signals, which required additional setup to receive the data. Other concepts for fracture healing monitoring did not use strain gauges, such as ultrasonic signals^17^, vibration analysis^18^, and fluid displacement^19,20^. Rajamanthrilage et al.^20^ proposed a technique using fluid displacement changes. The changes are caused by plate bending related to fracture healing. Unfortunately, most of the research has limitations preventing stepping up to clinical usage or commercialization, such as consisting of non-biocompatible material and requiring complex parts or apparatus.

As previous studies have proposed many practical concepts, we found that many devices either required special equipment or procedures to capture, transmit, or read the result. There is an opportunity for a new concept aiming to design a device that can monitor the fracture with accessible equipment while maintaining the biocompatible material for the potential to conduct clinical trials in future works. This research proposes a novel fixation plate with a sound generation mechanism for detecting bone union called a BUDWF plate. Our concept gives a quantitative result by monitoring the change in the wire’s natural frequency, which changes proportionally to bone restoration. The designed device is manufactured from biocompatible material while avoiding using electronic and complex elements to ensure biocompatibility and potentially conduct clinical trials shortly. Furthermore, the developed device can perform a noninvasive inspection without requiring any complex receiver or expensive medical equipment since a user can measure that sound with an ordinary microphone and a computer to perform the measurement.

## Methods

### Natural frequency of wire oscillation

Wire natural frequency related to the given tension. In string instruments, the wire must be set at a specific tension to create a proper natural frequency of the musical notes. Mersenne's law describes the frequency of the wire vibration^21^. The wire's natural frequency relates to the wire's tension (T), wire length (l), harmonic number (n), and linear density ($\rho )$ by Eq. (1)^22^. The benefit of using sound frequency is that the sound can easily and wirelessly transmit outside the human body, and there are diagnostics involving listening to the sound of internal organs. In addition, sound frequency is a property of a mechanical wave that remains constant when it travels to different mediums^23^, making it suitable as the measuring variable.

1 $$f= \frac{n}{2l}\sqrt{\frac{T}{\rho }}$$

### Design specification

#### Plate specification

The size of the fixation plate is a crucial design specification that affects the applications. The upper limit of plate length is under the average length of the tibia bone, which is around 40 cm^24^, and the average proximal width is approximately 4 cm^25,26^. The material for the plate is medical grade 316L Stainless steel for biocompatibility^27^.

#### Wire specification

The wire is made of 316L Stainless steel. Since this design used the wire's natural frequency to represent fracture, there are considerations in installing wire and designing a sound generation mechanism. The wire was intended to generate frequencies above 2000 Hz, which is more than the frequency range of the noises in hospitals^28,29^

#### Sound generation mechanism specification

Active electrically powered implants are a higher risk class in medical device classification than passive devices^30,31^. A higher risk class means higher harm may occur to the user if the device malfunctions or breaks. Our design aimed to avoid all electronic and complex elements using simple yet effective parts and mechanisms to reduce the risks. This design also reduces the difficulty in manufacturing and commercializing. PEEK was selected as a material due to its high young modulus and biocompatibility.

### Conceptual design

As the healing continues, the bone is slowly restored to its strength while the load on the fixation plate decreases over time in the healing process^11^. We propose a novel fixation plate attached to the wire to generate the sound and use the wire's natural frequency for detecting bone union. The wire's natural frequency corresponds to the wire tension and load on the fixation plate. We can implement this concept of detecting frequency changes due to changes in the load on the fixation plate. For explanation, when an external force is applied over a fractured bone with the fixation plate. The bone is unstable, so most of the load is distributed to the plate, which makes the plate highly bent, and the attached wire is tightened, increasing the wire's natural frequency. However, when applying force on union bone, the bone can carry most of the load, causing a small load to be distributed to the plate. The plate bending is minor in this case, and the tension on the wire decreases, resulting in a lower natural frequency.

### Detailed design

**Figure 1:**
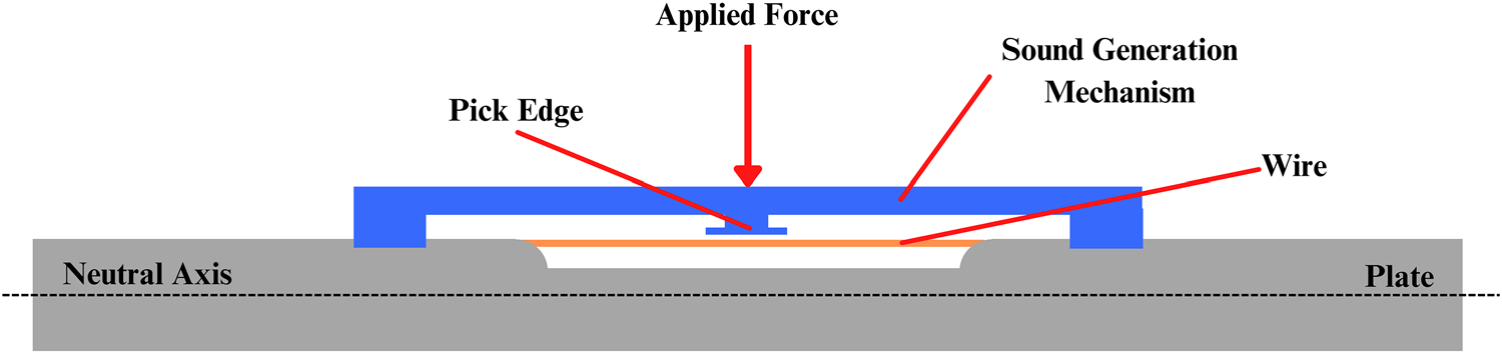
Conceptual design of the acoustic internal fixator.

**Figure 2:**
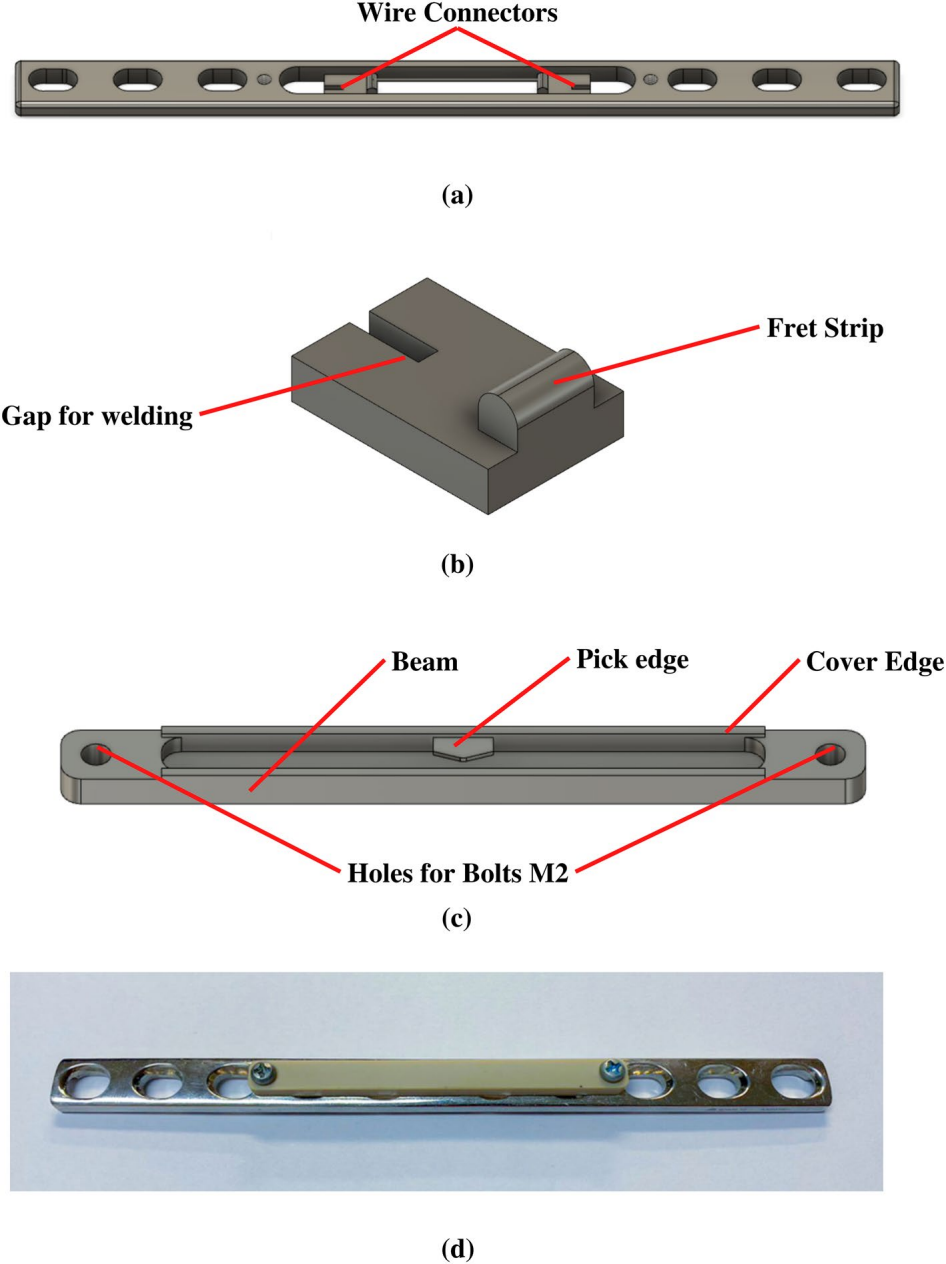
Plate design (**a**) Modified Plate (**b**) Wire Connector (**c**) Sound generation mechanism (bottom view) (**d**) Finished prototype of BUDWF Plate.

The bone union detection using a wire's natural frequency (BUDWF) plate consists of 3 main components: the internal fixation plate, the wire, and the sound generation mechanism, as shown in Figure 1. For the plate, the fixation plate is modified to have an open slot in the middle, and the 316L steel wire is laser welded on the surface of the plate. The sound-generating mechanism was created from a deformable beam, which is a simple structure. Then, add a pick edge, as shown in Figure 2c, to click the wire and generate sound. The physician can examine the fractured bone using this device by gently pressing the device from the outside. Additionally, the side cover edge of the mechanism has been slightly extruded to cover the inner part from soft tissue.

#### Plate and wire connector design

In this approach, we modified the commercial 10-hole fixation Syncera ADLER B0409.10 plate for installing wire and a sound generation mechanism. The commercial plate (dimension of 167 mm × 12 mm × 4 mm) was machined to create a 7 × 60 mm slot. The wire connector was designed to install and tighten the wire, as shown in Figure 2a. We designed the wire connector (Figure 2b) to have the exact width of the slot with a small gap at the end for welding the wire and added a small fret strip like the guitar fret. This fret strip is 3.5 mm in height to locate the wire above the neutral plate axis and acts as the end point of the wire vibration. The fret strip prevented the vibration at the weld point, minimizing the fatigue damage caused by the vibration. After machining, the wire and connectors were installed by laser welding. The wire was welded to the connectors first. Next, the connectors were pulled to tighten the wire until the desired frequency was reached. Then, the connectors were welded to the plate.

#### Sound generation mechanism design

Figure 2c shows the overall Sound generation mechanism (SGM). We designed a pick edge for hitting and actuating the wire. The 0.5 mm thick pick edge was installed in the middle of this structure. The mechanism was fabricated as a beam structure with a thickness of 1.5 mm. When the beam is pressed at the middle section by an external force, the midpoint of the beam is bent, allowing the pick edge to hit the wire to generate sound. Then, with the SGM's elasticity, the beam is returned to its original position and ready for the next activation. This mechanism is simple, easy to manufacture, and can mimic the motion of guitar strumming. Moreover, the last one, the mechanism can shield the inner parts from growing tissue on top of the plate; we included the cover edge by extruding the 0.5 mm thick wall at both sides of the beam. Lastly, two bolts attached the Sound generation mechanism to the top of the plate.

#### Wire design

The desired frequency should be set above the ambient frequency of the noise in the hospital, which is in the range of 500–2000 Hz^28,29^; we set the desired frequency at 3000 Hz to avoid the interference of the effect from ambient hospital frequency. Then, we varied other related parameters to meet the desired frequency. By considering Eq. (1), we reduced the length (l) and the mass per length of the wire ($\rho )$ by using the small wire diameter to increase the natural frequency. We selected the 0.3 mm diameter 316L stainless steel wire since it is the smallest medical wire available, which should minimize the diameter and its mass per length. B The wire was cut to 40 mm long. Then, we applied pretension to the wire to achieve a frequency of around 3000 Hz. It guarantees the sensor output is above the noise range since when the plate is bent, the tension from the bending is added up to the pretension, generating a sound above 3000 Hz.

## Experiment setup

**Figure 3:**
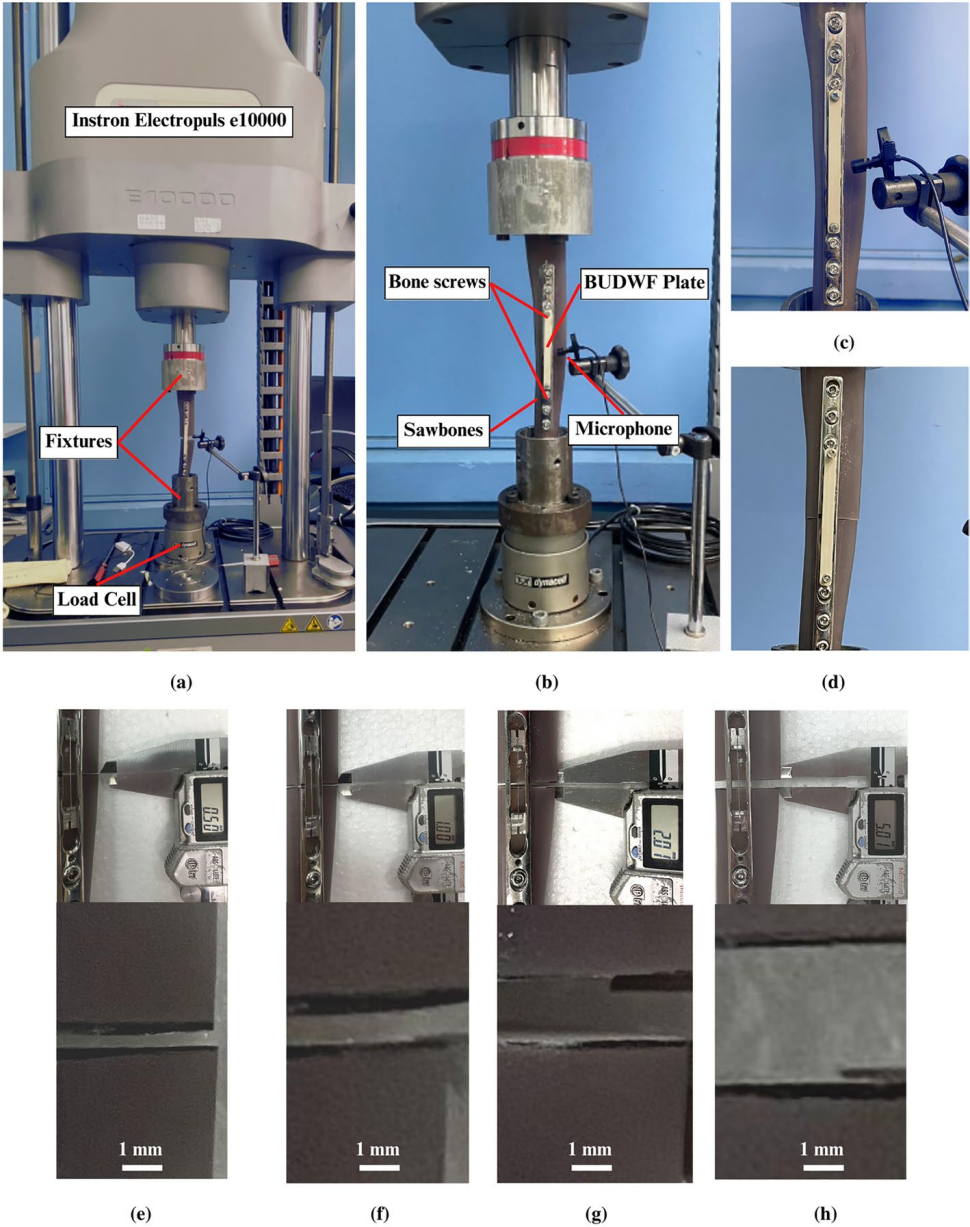
The experiment setup (**a**) The overall experiment setup (**b**) Close up of the setup with union sample (**c**) Close up of union sample (**d**) Close up of 0 mm gap fracture (**e**) Close up of 0.5 mm gap fracture sample (**f**) Close up of 1 mm gap fracture sample (**g**) Close up of 2 mm gap fracture sample (**h**) Close up of 5 mm gap fracture sample.

We conducted the experimentation aimed to investigate two experiments. The first experiment objective was to study the sensor output to be distinct from the ambient. The second was to examine the ability to detect the bone union by measuring the difference in the rate of the wire's natural frequency change from the fracture and union samples. Figure 3a and b provide an overview of the test rig. A Sawbones 3401 tibia composite mimics were used in these two experiments. BUDWF plate (Figure 2d) was installed on the Sawbones samples and tested on universal tester *Instron Electropuls e10000.* A Synco G1A1 condenser microphone was used to capture the generated sound. The specimens were prepared into six different samples. The uncut Sawbones represented the union bone as shown in Figure 3c. The fractured samples were prepared by cutting the Sawbones into five sizes (0, 0.5, 1, 2, and 5-mm gap) as shown in Figure 3d–h. The designed plate was fixed with six bone screws then samples were installed in the fixture at both ends. The condenser microphone was placed in the middle, 20 mm from the plate.

In the first experiment, we aimed to study the difference between the sensor output and the ambient sound. The ambient sound cannot interfere with the sensor output for the sensor's feasibility. So, the sensor must create a sound with frequency and amplitude distinct from the frequency range and amplitude of the ambient sound. The experiment was conducted in a quiet room. We studied the 5 mm fracture gap and union samples in this experiment. The samples were tested at 0 N and under compressive load 400N (estimated one-half body weight of patient 80 kg). Then, the sound generation mechanism was activated to generate sound. The spectrograms from both samples were plotted and compared with the ambient spectrogram.

In the next experiment, we aimed to study the device application when installed on different fracture sizes. We tested all six samples and compared the frequency at every load point from all six samples. The compressive force was progressively applied from 0 to 400N and decreased to 0 N at the same rate of 10N/s. We considered the loading cycle as the sample completes the set of increasing and decreasing loads. The wire frequencies were measured at 0, 100, 200, 300, and 400N in increasing and decreasing loading cycles. The sound generation mechanism was actuated ten times for each load point, and then the average frequency was plotted to find the slope representing the rate of frequency change from both samples. Then, we repeated the experiment five times to study the repeatability.

## Results

### Difference between the sensor output and the ambient sound

**Figure 4:**
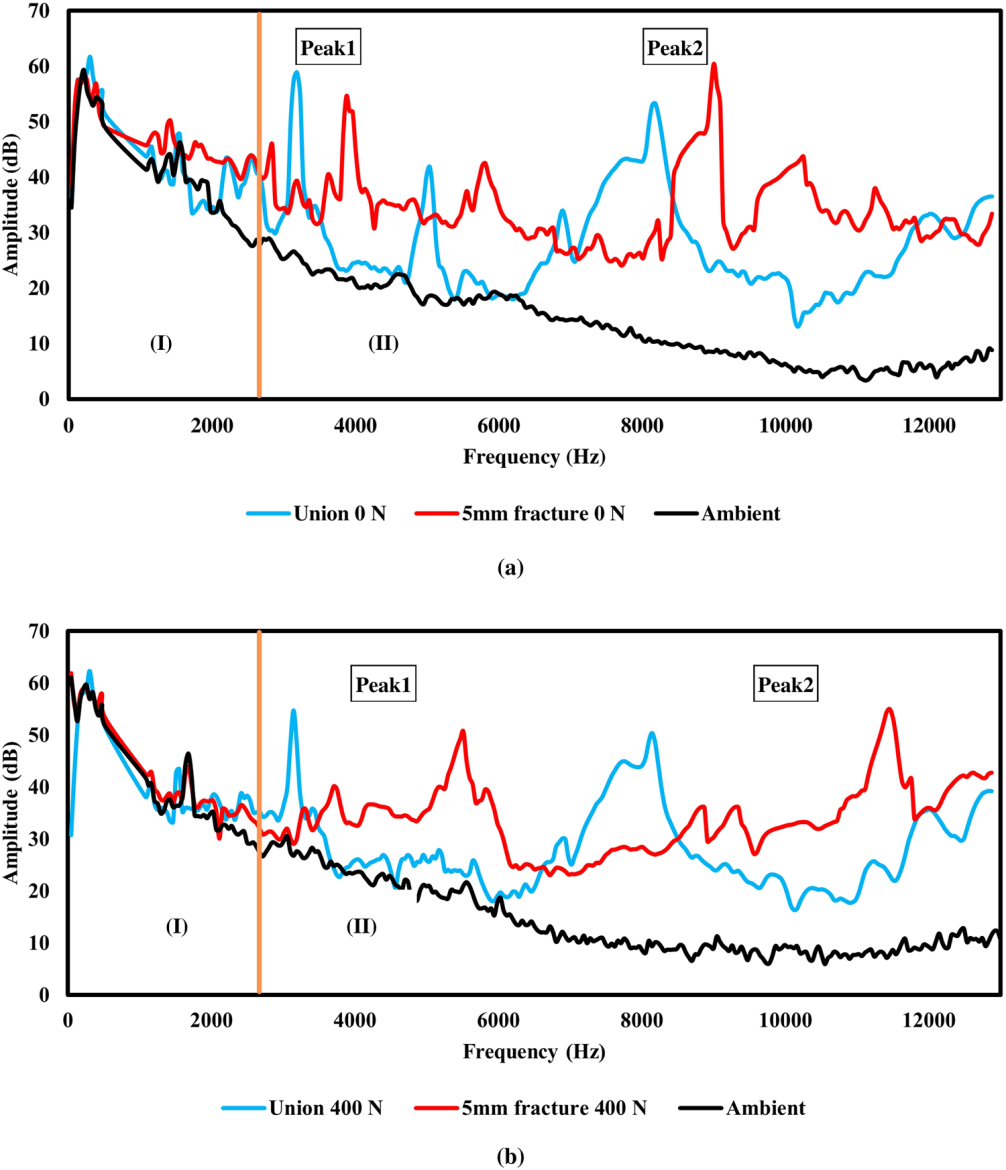
Spectrogram of sounds (**a**) compared signals from fractured and union samples at 0 N and (**b**) compared signals from fractured and union samples at 400 N.

The first experiment found the difference in the sound spectrogram between ambient and generated sound, as shown in Figure 4a and b. The black line represented the spectrogram of ambient sound, while the red and blue lines represented the sound from fractured and union samples, respectively. All three lines shared similar patterns and amplitudes below 3000 Hz (zone I). On the other hand, considering the frequency range above 3000 Hz (zone II), the red and blue lines differed from the black line as both generated sounds have higher amplitude than the ambient. We found two frequencies with high amplitude and distinguished them from others, defining Peak 1 as the lower frequency and Peak 2 as the higher frequency. When considering every Peak 1 and 2 in spectrograms from both samples in every load point, we found that the fractured sample always has a higher frequency than the union sample.

### Detecting the bone union

**Figure 5:**
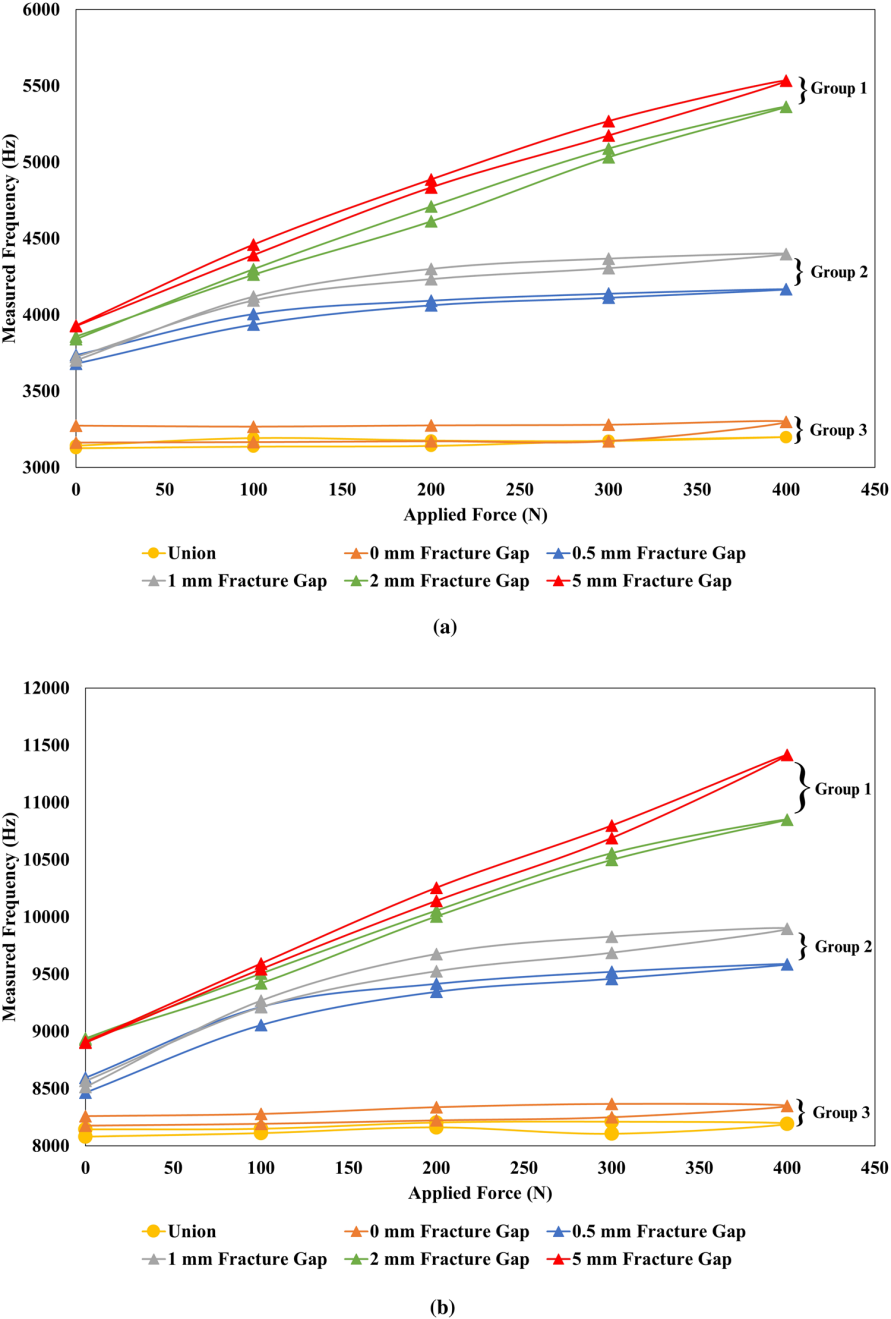
(**a**) Measured frequencies at Peak 1 from five loading cycles at all load points from every test sample (**b**) Measured frequencies at Peak 2 from five loading cycles at all load points from every test sample.

We investigated the frequency changes in all six samples. The natural frequencies from five cycle tests at all load points were plotted as shown in Figure 5a for Peak 1 and Figure 5b for Peak 2. The lines represented the five cycles' average frequencies from 5 samples as 5 mm gap fracture sample (red), 2 mm gap fracture sample (green), 1 mm gap fracture sample (grey), 0.5 mm gap fracture sample (blue), 0 mm fracture gap (orange), and union sample (blue). We found that the rates of frequency changes (slope) from each sample were different, and they can be categorized into three groups.

In group 1 (2 mm and 5 mm fracture gap), the rates of frequency change from these samples were significantly high when compared to other groups. For a 2 mm fracture gap sample, the average rates of frequency change were 3.86 ± 0.11 Hz/N for Peak 1 and 5.03 ± 0.28 Hz/N for Peak 2. For a 5 mm fracture gap sample, the average rates of frequency change were 4.10 ± 0.20 Hz/N for Peak 1 and 6.18 ± 0.27 Hz/N for Peak 2.

**Figure 6:**
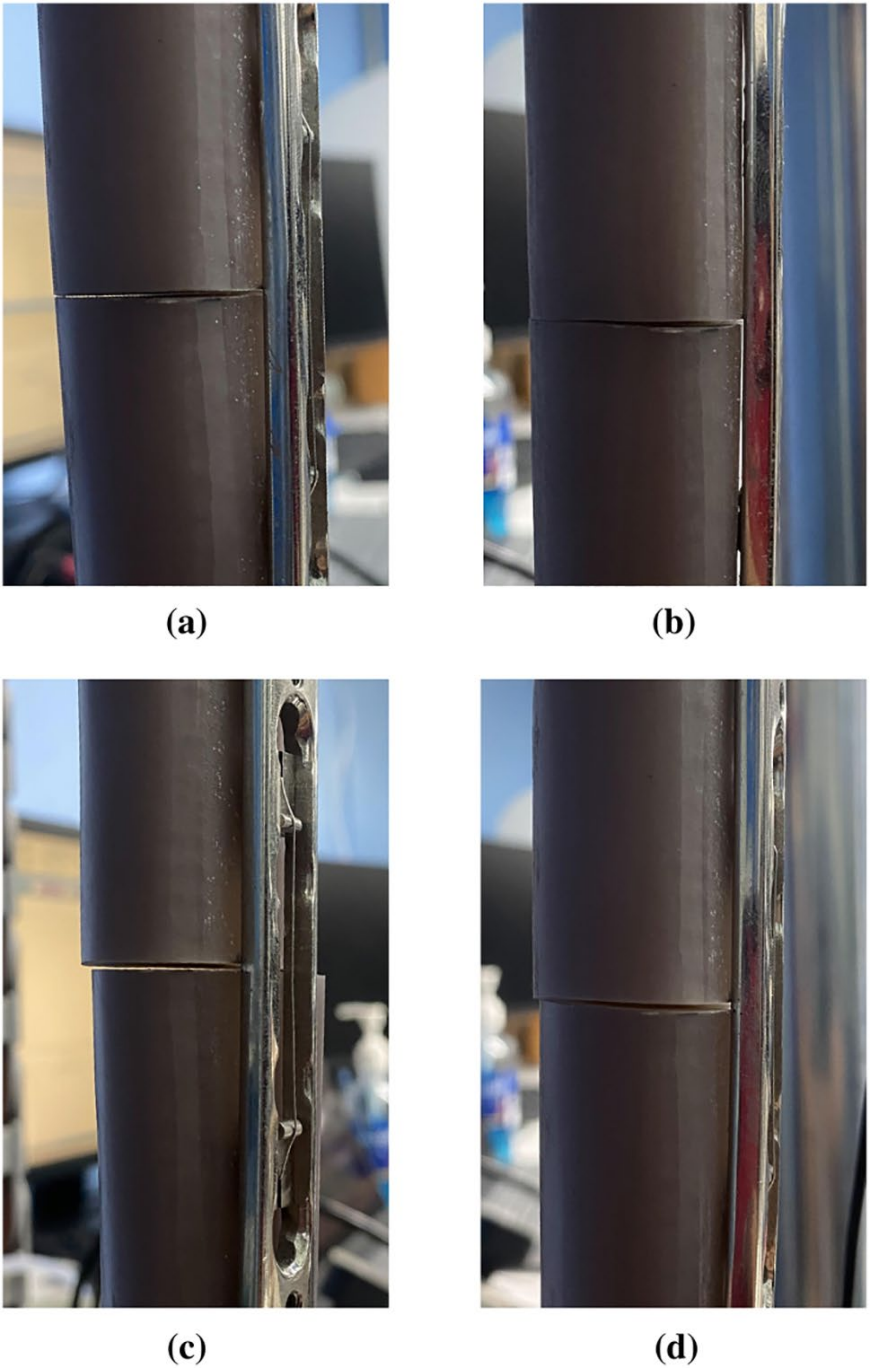
(**a**) 0.5 mm fracture gap sample at 0 N (**b**) 0.5 mm fracture gap at 100 N where the gap was closed (**c**) 1 mm fracture gap at 0 N (**d**) 1 mm fracture gap at 100 N where the gap was closed.

In group 2 (0.5 mm and 1 mm fracture gap), the rates of frequency change were changed in 2 stages. The first stage had high rates of frequency change, and then the rates of frequency change were close to zero after the applied force reached above 100 N. Our observation found that the separated samples were in contact at 100 N. Figure 6a shows the 0.5 mm fracture gap sample before the samples were contacted, and Figure 6b shows the 0.5 mm fracture gap sample after 100 N where the samples were contacted. For the 0.5 mm fracture gap, the average rates of frequency change at 0–100 N were 2.60 ± 0.39 Hz/N for Peak 1 and 5.98 ± 0.91 Hz/N for Peak 2, and 0.51 ± 0.14 Hz/N for Peak 1 and 0.57 ± 0.12 Hz/N for Peak 2 at 100–400 N. In a 1 mm fracture gap, the average rates of frequency change at 0–100 N were 2.99 ± 0.24 Hz/N for Peak 1, 5.80 ± 0.55 Hz/N for Peak 2, and 0.37 ± 0.13 Hz/N for Peak 1 and 0.74 ± 0.25 Hz/N for Peak 2 at 100–400 N. Figure 6c shows the 0.5 mm fracture gap sample before the samples were contacted and Figure 6d shows the 0.5 mm fracture gap sample after the samples were contacted.

In group 3 (union and 0 mm fracture gap), the rates of frequency change from union and 0 mm fracture gap were near zero. For the union sample, the average rates of frequency change were 0.13 ± 0.03 Hz/N at Peak 1 and 0.19 ± 0.04 Hz/N at Peak 2. For a 0 mm fracture gap sample, the average rates of frequency change were 0.13 ± 0.01 Hz/N for Peak 1 and 0.33 ± 0.04 Hz/N for Peak 2.

## Discussion

### Simplicity for manufacturing and usage

The bone union detection via a natural wire frequency osteosynthesis plate (BUDWF plate) consisted of 3 components that were biocompatible and easy to manufacture as the medical grade wire was commercially available, and the plate and sound generation mechanism was created using biocompatible materials with simple geometry. In usage, this device can easily operate when compared to other studies. This device plate does not require other complex equipment, such as X-ray image^20^, ultrasound^19^, and data receiver^16,17^. This device can perform a noninvasive measurement by measuring the sound created by the wire on the plate. The required instruments for measurement were a condenser microphone and a computer with sound analysis software, which are commonly available.

### Performance

The natural frequency from the BUDWF plate is mixed with the ambiance frequency, making them unsuitable to use in the frequency range below 3000 Hz. As shown in Figure 4a and b, there are similarities in the pattern of the spectrograms in the frequency range of 0–3000 Hz due to the sound from the laboratory equipment. However, the difference in amplitude between generated sound spectrograms and ambient was more significant in the high-frequency range. At above 3000 Hz, we found that the generated sounds have higher amplitude and different patterns. This difference can prevent any misinterpretation of ambient noise from experimentation.

Figure 5a and b illustrate the rates of frequency change at all load points. As we mentioned in the results section, We found three different slope patterns that can be explained by Mersenne's law^22^.

In group 1 (2 and 5 mm fracture gap), we found that the rates of frequency change were high. This is because when the applied force increases on these fracture samples, most of the applied force is on the plate, causing the plate to bend, leading to higher wire tension and a highly increased natural frequency of wire of the fractured samples.

In group 2 (0.5 and 1 mm fracture gap), we found two different rates of frequency change. When the fractured samples were still separated, the rates of frequency change were high due to the high applied force on the plate and high plate bending. However, after the fracture samples were in contact, the applied force was distributed to the plate and the contact bone. As the compressive load was increased, the increased load was distributed to the contacted bone, resulting in low force applied on a plate and low plate bending, causing a low rate of frequency change.

In group 3 (union and 0 mm fracture gap), the rates of frequency change from the union sample were near zero even if the applied force increased. This is because most of the applied force is distributed to the bone, resulting in low bending on the fixation plate and lower wire tension. The 0 mm gap also had rates of frequency change near zero because the 0 mm gap fractured sample was still in contact. The applied force can be distributed like the union sample, resulting in low force, load bending on the plate, and low frequency change. We also found unequal frequencies at the starting point (0N) between groups 1, 2, and 3, as shown in Figure 5a and b. This was due to bending from installation. In group 3, the BUDWF was bent to fit the Sawbones geometry. This bending was in the direction that decreased wire tension, causing the lower frequencies at 0 N.

When considering the ability to differentiate fracture samples and the union sample, in group 1 (2 and 5 mm fracture gap), the BUDWF was able to distinguish 2 and 5 mm fracture gap samples from the union sample. The rates of frequency change were high on these fracture gap samples but were close to zero on the union sample. The differences between rates of frequency change from 2 to 5 mm fracture gap samples and union were statistically significant (Mann-Whitney, *p* = 0.012).

In smaller fracture gap samples (group 2), the fracture samples were contacted under an applied load greater than 100 N. Before the fracture bones were in contact, the differences in rates of frequency change from 0.5 to 1 mm fracture gap samples and the union sample were statistically significant (Mann-Whitney, *p* = 0.012). After the fractures were contacted, the differences in the rates of frequency change were not statistically significant (Mann-Whitney, *p* = 0.14). Despite the non-statistically significant differences, the pattern of the frequency graphs, as shown in Figure 5a and b, differed from the union sample.

In union and fractures without gap (group 3), the BUDWF cannot differentiate between the 0 mm fracture gap and the union bone because the 0 mm fracture gap sample distributes load similar to the union sample, causing the rate of frequency change from both scenarios to be near zero. The difference rates of frequency from fractures without gap and union were not statistically significant (Mann-Whitney, *p* = 0.061).

When comparing our results to other methods for detecting bone union in previous studies, the BUDWF can differentiate the fractures with smaller gaps than some previous methods. Mcgilvray et al.^16^ device successfully distinguished non-union sample with gap above 10 mm and the union sample. In the Rajamanthrilage et al.^20^ experiments, their device can differentiate the 5 mm gap and union samples. The BUDWF can differentiate the sample with a fracture gap above 2 mm from the union sample. Furthermore, in gaps smaller than 2 mm, the BUDWF had the potential to differentiate the fracture gap samples below 2 mm from the union sample as the pattern of the rates of frequency was visually different. There were statistically significant differences between the 0.5 and 1 mm fracture gap samples and the union sample before the fractures were contacted.

## Limitations and future works

In this study, we only experimented and discussed the results from the Sawbones specimens. There is a limitation as neither of the experiments was affected by the presence of liquid and soft tissue within the human body. The liquid and soft tissue could reduce the sound amplitude created by the wire. We suggest conducting an experiment to study the reduction in the BUDWF output with the influence of liquid and soft tissue in the future.

The BUDWF can differentiate between the fractures with a fracture gap above 2 mm and the union sample. This could be the first step to developing a bone union detection device using a wire's natural frequency. However, the BUDWF could not differentiate between fractures with a 0 mm fracture gap and union sample, as the load distribution on the plate and bone were nearly identical in these two scenarios. This is a limitation of the BUDWF, and we recommend avoiding using the BUDWF on contact fractures.

In addition, the results were insufficient to provide a threshold to determine the bone union. As in real scenarios, other parameters will affect device performance, such as the different types of fractures and the callus formation stages that change the mechanical properties of the fractured bone. To determine the threshold of the bone union, the BUDWF should be experimented in vivo to study its performance throughout the fracture healing process in the future.

The plate was designed specifically for the tibia, which is located near the skin. Our method for activating the sound generation mechanism requires external pressing force, which cannot be done if the device is deep in the human body. In future work, we suggest designing a sound generation mechanism that can be activated without applying direct force to the sound generation mechanism. Furthermore, the next development stage should aim to develop seal parts that completely prevent the effect of internal fluid and tissue that may interfere with the wire and sound generation mechanism.

## Conclusions

This study developed a novel osteosynthesis plate with bone union detection using a wire's natural frequency (BUDWF) and experimented with a Sawbones sample. The preliminary results show that the device can differentiate between samples with a fracture gap above 2 mm and a union sample. The concept of the device is measuring the wire's natural frequency change of the generated sound, which is proportional to the load applied on the bone following Mersenne's law. The plate can create a sound above 3000 Hz outside the ambient sound range. The device can differentiate fracture and union samples from the difference in rates of frequency changes. We found statistically significant differences in rates of frequency change between samples with a fracture gap above 2 mm and a union sample. The BUDWF does not use complex elements to operate. It requires only a microphone and computer to collect and analyze the results. With further developments and evaluations, this novel device could provide an alternative and convenient diagnostic tool for bone union detection.

## Data Availability

The data supporting this study's findings are available from the corresponding author (P.T.) upon reasonable request.
